# Thicker Carotid Intima Media Thickness in Children with Monocyte Chemoattractant Protein-1: A-2138T and A-2464G Mutation

**DOI:** 10.1155/2014/176535

**Published:** 2014-04-22

**Authors:** Yuyun Yueniwati, Valentina Yurina, Mohammad Rasjad Indra

**Affiliations:** ^1^Radiology Department, Medical Faculty, University of Brawijaya, Malang 65145, Indonesia; ^2^Pharmacy Study Program, Medical Faculty, University of Brawijaya, Malang 65145, Indonesia; ^3^Physiology Laboratory, Medical Faculty, University of Brawijaya, Malang 65145, Indonesia

## Abstract

Carotid intima media thickness (CIMT) is clearly associated with atherosclerosis. Studies in ischemic stroke patients reveal that there is a significant association between CIMT with monocyte chemoattractant protein-1 (MCP-1) and osteopontin (OPN) promoter polymorphism. This research aims to explain the effect of MCP-1 and OPN promoter polymorphism toward CIMT changes identified in Javanese Indonesian children. Subjects were 54 children: 27 were from parents with ischemic stroke (cases), and 27 were from healthy parents (controlled). The CIMT was examined by utilizing high resolution B-mode ultrasound. Physical examination and genotyping analysis of MCP-1 promoter were conducted by employing PCR method. Research results indicate that two polymorphisms were obtained, that is, A-2138T and G-2464A, respectively. A-2138T polymorphism was found in 5% of case children and in 14.3% of controlled children. G-2464A polymorphism was found in 5% of case children. CIMT of case children was significantly different from that of controlled children (0.61 ± 0.012 mm versus, 0.52 ± 0.015 mm, *P* = 0.021). Subjects with MCP-1 promoter polymorphism have 1.471 times higher tendency to have thicker CIMT than subjects with no polymorphism in MCP1 promoter. OPN promoter T-66G was also studied but it did not indicate occurrence of polymorphism in samples.

## 1. Introduction

Stroke is the second highest cause of death in the world and the main cause of disability in people in productive ages [1]. In Indonesia, the average age of stroke patients is 58.8 years old and stroke is the leading cause of high mortality rate in people above 50 years old. Number of stroke patients in productive age is increasing in recent decade [2]. This fact has close affinity with data reported by American Heart Association. Statistics of Heart Disease and Stroke—2013 Update reveals that number of stroke patients aged between 20–45 years has increased significantly in recent years [3].

According to World Health Organization, stroke is rapidly developing clinical signs of focal (or global) disturbance of cerebral function. The symptoms last in 24 hours or longer and may lead to death, with no apparent cause other than of vascular origin [1]. There are two types of stroke: ischemic and hemorrhagic. More than 87% of stroke cases are caused by ischemia due to thrombosis or cardio embolisms. The remaining 13% are due to hemorrhagic. Ischemic stroke is atherosclerosis-associated complication [3–5] which is progressive in process. It requires 3 to 4 decades or more since the period of endothelial dysfunction and carotid intimamedia thickness (CIMT) to develop until clinical manifestations appear [6]. An atherosclerotic process that begins in childhood will cause clinical manifestations in adulthood. A study on American children aged 10–14 years old who died from motorcycle accidents showed that 50% of them have early atherosclerosis evidence. Similarly, Bogalusa Heart Study revealed that 50% of its 204 subjects aged 2–15 years old had fatty streak in their coronary arteries [7]. An atherosclerosis which occurs in younger age shows worse prognosis [8]. Thus, detecting atherosclerotic vascular changes in childhood helps identifying high-risk groups which is very useful for the early stroke management [9].

CIMT is a surrogate marker that reveals atherosclerosis in general and constitutes a predictor of blood vessels condition in the future [10]. The increase of CIMT has been proven to be related with the raise of ischemia stroke risk [11–13]. In Indonesia, CIMT measurement has not yet become a routine screening procedure for atherosclerosis prevention. To date, there is no normal value data of CIMT for population in Indonesia.

The atherosclerotic incidence is contributed significantly by genetic factors. CIMT increases in the early progession of atheroslerosis. As a result, this sign becomes very useful to be intermediate phenotype in genetic studies [14, 15]. Epidemiological data show that the increase of CIMT is influenced by genetic factors, with the prediction that 30% to 40% is heritable [12].

In 2006, GENetique de l'Infarctus Cerebral [GENIC] Study conducted a research on genetic factors related with CIMT of cerebral infraction patients. The study examined polymorphism association with vascular pathologies which incorporate inflammation, hypertension, coagulation, and lipid metabolism. The findings revealed that there was a significant relationship between CIMT with monocyte chemoattractant protein-1 [MCP-1] and osteopontin (OPN) polymorphisms [12].

MCP-1 is a preinflammatory cytokine which works on blood vessel endothelium changes [10]. MCP-1 is synthesized by various cells associated with atherosclerosis, including endothelial cells, muscle cells, fibroblasts, and macrophages. In early stages of atherogenesis, MCP-1 is primary chemokine which recruits monocytes into arterial subendothelium. MCP-1 protein and RNA are highly evidenced in atherosclerotic vessels, but not in normal vessels [12]. The MCP-1 polymorphism in the forms of SNP-957, SNP-2518, and SNP-2578 causes earlier occurrence of CIMT faster progression [12, 16–19] in Chinese, Japanese, French, and Slovakian people. However, the mechanism of earlier and faster CIMT development could not be identified yet [12]. Some researchers suggest that ethnic and geographic factors give different effects on genetic polymorphisms taking in MCP-1 polymorphism [19]. Thus far, there has never been a study on MCP-1 polymorphism and its contribution toward CIMT conducted for Indonesian population.

OPN is a multifunctional protein expressed by various cells. It functions in atherosclerosis, in cell-mediated immunity, and in macrophage recruitment and activation. In early stages of atherosclerosis, OPN attracts inflammatory cells, promotes the releases of proteinolytic enzymes, and stimulates smooth muscle cell proliferation [20]. There is a strong relationship between CIMT and OPN promoter polymorphism. The OPN promoter polymorphism is studied in several areas, such as in SNP-443 and SNP-66 [12, 21]. This research attempts at explaining the effect of MCP-1 and OPN promoter polymorphism on CIMT changes in Javanese Indonesian children.

## 2. Methods

### 2.1. Subjects and Blood Collection

The research design is case control analytic observation. Subjects were 54 Javanese Indonesian children who were classified into two groups: case and controlled. The case group consisted of 27 children aged 10–21 years whose parents had ischemic stroke historical background. The controlled group consisted of 27 Javanese children aged 10–21 years whose parents had no ischemic stroke history. All parents had general characteristics: 40–50 years old, nonsmoking, and nonobese. All participants were required to complete a standardized questionnaire designed to obtain information about family history. Blood samples were collected after 10–12 hours fasting through venous puncture. Plasma concentrations of total cholesterol, high-density lipoprotein [HDL], low-density lipoprotein [LDL] cholesterol concentrations, triglycerides, and fasting blood glucose were measured in accordance with standardized protocols in the Physiology Laboratory of Medical Faculty, University of Brawijaya Malang. The research had been approved to be conducted by Ethical Committee, Medical Faculty, University of Brawijaya, Malang, Indonesia.

### 2.2. High Resolution Carotid Ultrasound

**Figure 1:**
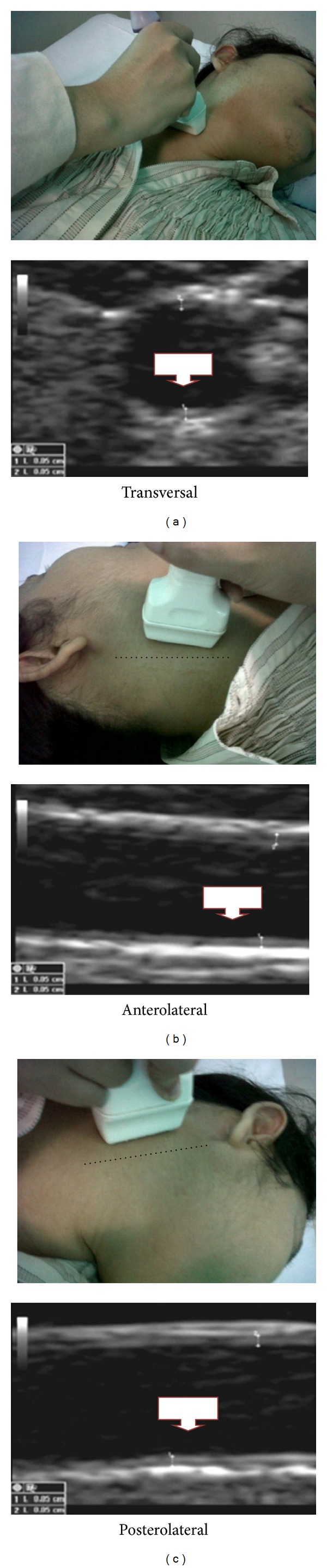
Observation angles for CIMT measurement: (a) transversal, (b) anterolateral, and (c) posterolateral. The dashed lines represent the sternocleidomastoid muscle.

To measure CIMT, all subjects went through carotid ultrasound process in the Radiology Department of Saiful Anwar General Hospital in Malang, Indonesia. High resolution carotid ultrasound measurements were performed using LOGIQ S6 Ultrasound (GE Healthcare), with 10 MHz linier transducer. The measurement was done by scanning the far wall of common carotid arteries in 1.0 cm distal. The crest at the origin of the bifurcation was used as an anatomical mark to identify segment to be visualized. In each examination, the sonographer used three different scanning angles, namely, transversal, anterolateral, and posterolateral angles, to record the greatest CIMT (Figure 1) [22–24].

### 2.3. Genotyping Analysis

Genomic DNA was isolated from 1 mL blood using extraction kit (Wizard Genomic DNA purification Kit/Promega). The isolated DNA was analyzed by using 1% agarose gel electrophoresis. MCP and OPN promoters were amplified by using specific primer MCP forward 5′-CCGAGATGTTCCCAGCACAG-3′ and MCP reverse: 5′-CTGCTTTGCTTGTGCCTCTT-3′ [16]. OPN forward 5′ATTACAATTCGTGACTGCCTGCC3′ OPN reverse 5′TGTACCTTGGTCGGCGTTTG3′ [25]. Amplified DNA was sequenced by employing automated sequencer (Macrogen, Seoul, Korea). Sequencing result was analyzed by using SeqMan (DNASTAR) program.

### 2.4. Statistical Analysis

Data were analyzed by using descriptive and inferential statistical method. Data characteristics, laboratory test results and ultrasound results were tested with Data Normality Test (Kolmogorov-Smirnov) and Data Homogeneity Test (Levence Statistics) prior to comparison test by using independent* t*-test. The calculation of mutation influence on carotid artery toward CIMT-increasing risks was done using the odd ratio [OR] in a 2 × 2 table.

## 3. Results

### 3.1. Carotid Intimamedia Thickness Measurement

Each sample received the same treatment in which right and left common carotid arteries were checked, and CIMT and diameter of the carotid artery were measured and calculated.

Results of the ultrasound examination appear in Table 1.

Result of correlation analysis on the average CIMT with clinical characteristic variables is presented as in Table 2.

Result of correlation test carried on using Pearson Product Moment test showed that there was no correlation between average CIMT with age, height, weight, body mass index, fasting glucose, total cholesterol, HDL, and LDL, as well as triglycerides.  *P*  values are greater than  *α* = 0.05  for all parameters.

### 3.2. Genotyping Analysis on Monocyte Chemo Attractant Protein-1 (MCP-1)

**Figure 2:**
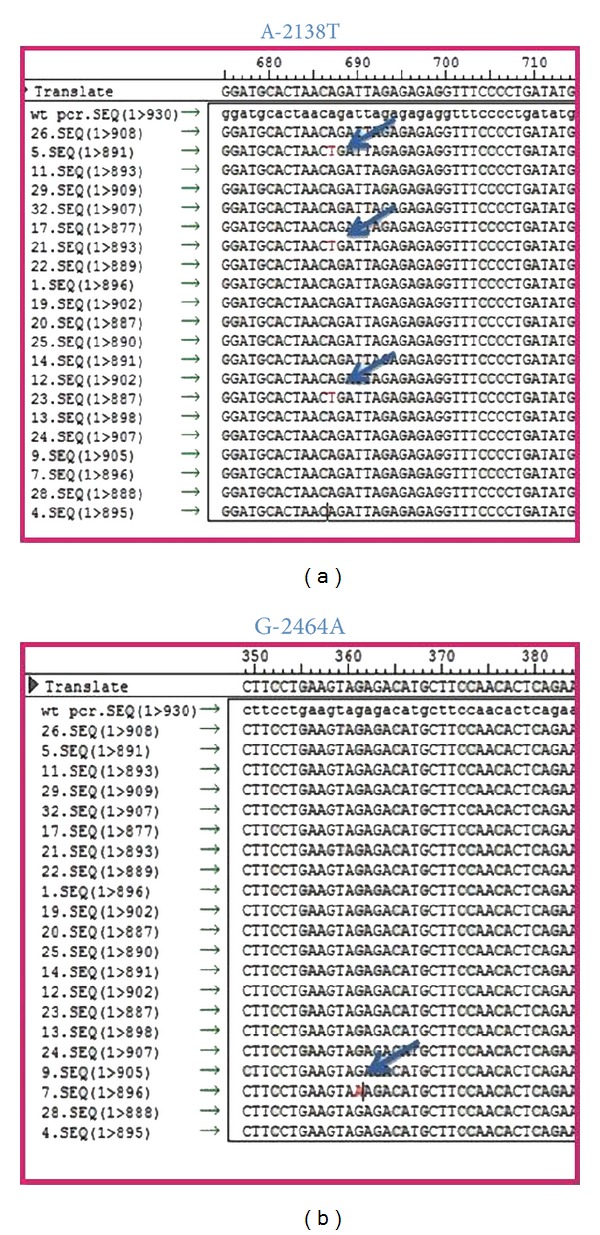
Mutation analysis results using Seqman program (DNASTAR): (a) A-2138T mutation occurs in two controlled samples and one case sample; (b) A-2464G mutation occurs in one case sample; mutation is shown by arrow.

Mutations took place in base number-2138 in which adenine base had substituted to thymine base. The mutations occurred in two controlled samples (14.3%) and one case sample (5%) (Figure 2). Further mutations took place in base number-2464 in which guanine base mutated into adenine base. The mutations occurred in a single case sample (5%).

The calculation of mutation influence on CIMT-increasing risks measured through odd ratio [OR] using a 2 × 2 table appears as in Table 3.

Based on previous research [11, 24, 26], 0.5 mm was set as the normal value of CIMT for odd ratio analysis. The odd ratio of mutation on CIMT is OR = 1.471[1.216 − 1.779]. This means that children with mutation have 1.471 times higher tendency of having CIMT thickening than children without mutation.

### 3.3. Genotyping Analysis on Osteopontin (OPN)

All samples were analyzed for identifying T-66G OPN polymorphism promoter, but there was no mutation found in all samples.

## 4. Discussion

From the research, two types of point mutation in MCP-1 gene promoters were found, that is, A-2138T and G-2464A. Either base-2138 or base-2464 was located in promoter site. The promoter determines the efficiency of RNA polymerase binding and, thus, it also determines the transcription efficiency. The transcription factor is attached to the binding site in the promoter region and stimulates RNA polymerase to bind with the promoter site. Polymorphism on the promoter site will affect the transcriptional activity and gene expression [27].

In Japan, MCP-1A-2138T polymorphism has been reported to be identified in myocardial infarction patients above 65 years old. It was found out that the MCP-1 A-2138T polymorphism was significantly associated with MCP-1 serum level and incidence of myocardial infarction [28]. In this research, MCP-1A-2138T polymorphism was found in children from parents with ischemic stroke history and children whose parents do not have ischemic stroke history as well. The polymorphism has never been reported to be found in people with no ischemic stroke history previously. Therefore, our finding is new.

Children with A-2138T polymorphism, either from the case or the controlled group, had a thicker CIMT than the normal value. The two children with A-2138T polymorphisms had a family history of myocardial infarction disease with body mass index over 25. Research conducted by Iwai et al. (2006) informed that in Japan A-2138T polymorphism was found in patients with body mass index of more than 25 kg/m^2^ with myocardial infarction [28].

MCP-1 G-2464A polymorphism is also located on the promoter site. In this research, the polymorphism was found in the case sample of a 19-year-old girl who inherited it from the mother suffering from ischemic stroke at the age of 32. Her grandfather also suffered from ischemic stroke with hypertension, while her grandmother suffered from hypertension. Her total cholesterol was 208 mg/dL and LDL cholesterol was 136 mg/dL, which indicated dyslipidemia. Her average CIMT was 0.60 mm, or thicker than normal average CIMT [11, 26].

Results of this research reveal that children with polymorphism had thicker CIMT than children without mutation. Risk measured with odd ratio shows that children with MCP-1 polymorphism promoter had 1.471 times possibility to have thicker CIMT than children without polymorphism. These results confirm the importance of genetic examination, especially the MCP-1 polymorphism promoter analysis in children with atherosclerosis risk factors. The presence of polymorphism indicates the importance of preventive treatment in early atherosclerotic management.

MCP-1 polymorphism promoter increases the MCP-1 expression level. MCP-1 works synergistically with its receptor, CC Chemokine Receptor 2 (CCR2). Result of this interaction is MCP-1/CCR2 messenger system increasing activity which leads to the local recruitment of monocytes at the site of injury in the arterial wall. This, in turn, will lead to increasing atherosclerosis incidence [29, 30].

The relation of MCP-1 promoter polymorphism with increasing CIMT has been reported in several studies, in which the MCP-1 G-927C and A-2578G polymorphisms are significantly associated with the increasing CIMT in patients with ischemic stroke [12]. The MCP-1 G-362C polymorphism in black population is associated with the increasing CIMT and atherosclerosis risk [29]. The MCP-1 G-928C polymorphism is associated with the increased CIMT and the atherosclerosis risk [29, 30]. In contrast to some other studies conducted on atherosclerosis-related conditions, this research did not result in any finding on MCP-1 A-2518G polymorphism [16, 31].

The difference among the above genetic polymorphism findings is likely to be due to the ethnic and geographical differences [19]. Data on the Indonesian polymorphism, including MCP-1 gene polymorphism, is lacking. High MCP-1 transcription rate contributes to the severity of stroke [32, 33]. The MCP-1 transcription rate is influenced by several risk factors such as hypertension, hypercholesterolemia, smoking habit, and diabetes [29].

## 5. Conclusion

In summary, two MCP-1 promoter polymorphisms were found in the research, namely, A-2138T and G-2464A. Children with polymorphism demonstrate increasing CIMT and have 1.471 times higher possibility to have thicker CIMT. CIMT of Javanese Indonesian children from parents with ischemic stroke is thicker than CIMT of children from healthy parents.

## Figures and Tables

**Table 1:** Ultrasound examination results.

	Case (*n* = 27)	Control (*n* = 27)	*P* value

Mean of the right IMT (mm)	0.61 (0.015)	0.53 (0.016)	0.032
Mean of the left IMT (mm)	0.60 (0.013)	0.51 (0.018)	0.034
Mean of the right and left IMT (mm)	0.61 (0.012)	0.52 (0.015)	0.021
Mean of the right carotid diameter (mm)	6.38 (0.058)	6.51 (0.051)	0.397
Mean of the left carotid diameter (mm)	6.29 (0.047)	6.57 (0.047)	0.034

Independent Samples *t*-tests. Data are mean (SD).

**Table 2:** Average CIMT Correlation Analysis.

Independent variables	Correlation coefficient	*P* value
Age	−0.010	0.943
Height	0.112	0.422
Weight	0.098	0.482
Body mass index	0.046	0.739
Systolic blood pressure	−0.028	0.839
Diastolic blood pressure	0.062	0.654
Fasting glucose	−0.053	0.705
Total cholesterol	0.168	0.226
LDL cholesterol	0.253	0.065
HDL cholesterol	−0.149	0.282
Triglycerides	0.205	0.137

Dependent variable = CIMT.

**Table 3:** 

	CIMT ≥ 0.5 mm	CIMT < 0.5 mm	Total
Mutation [+]	4	0	4
Mutation [−]	34	16	50

Total	38	16	54
